# Nurse Leaders' Perceptions of Development of Their Own Interpersonal Communication Competence: A Qualitative Descriptive Study in Social and Healthcare Organisations

**DOI:** 10.1111/jan.70281

**Published:** 2025-10-15

**Authors:** Petra Kämäräinen, Leena Mikkola, Anu Nurmeksela, Tarja Kvist

**Affiliations:** ^1^ Department of Nursing Science Faculty of Health Sciences, University of Eastern Finland Kuopio Finland; ^2^ Department of Communication Sciences Faculty of Information Technology and Communication Sciences, Tampere University Tampere Finland; ^3^ Wellbeing Services County of Central Finland Jyväskylä Finland

**Keywords:** interpersonal communication, leadership, management, professional development, qualitative approaches

## Abstract

**Aim(s):**

To describe nurse leaders' perceptions of factors related to the development of their own interpersonal communication competence.

**Design:**

Qualitative descriptive study.

**Methods:**

Individual semi–structured interviews were conducted with 21 nurse leaders in three wellbeing service counties in Finland. Data were collected between February and April 2024 and analysed using an inductive content analysis.

**Results:**

The analysis identified two main categories, each comprising several subcategories: (1) *individual factors related to development,* which encompassed participants' perceptions on how inherent qualities, personal experiences, reflexivity, motivation and communication training were related to the development of interpersonal communication competence, and (2) *interpersonal factors related to development*, which demonstrated the role of situational contexts, feedback and social support in competence development.

**Conclusion:**

This study frames the development of nurse leaders' interpersonal communication competence as a lifelong and continuous process. The findings emphasise the interplay of individual and interpersonal factors, encompassing various competence categories and dimensions. This dynamic underscores the importance of developing interpersonal communication competence in workplaces through various practices, in addition to formal communication training.

**Implication for the Profession:**

The findings enhance our understanding of nurse leaders' interpersonal communication competence and the factors related to it. The study also identifies various practices for fostering nurse leaders' communication competence.

**Impact:**

The study provides valuable insights for healthcare organisations and educational institutions by highlighting the importance of providing nurse leaders with opportunities to promote their interpersonal communication competence through both formal communication training and their daily work activities.

**Reporting Method:**

Reporting adhered to the SRQR checklist.

**Patient or Public Contribution:**

No patient or public Involvement.

**Summary**

- What does this paper contribute to the wider global clinical community?
  ○ This paper offers novel insights into how nurse leaders perceive the development of interpersonal competence and the factors they consider important in competence development, thereby contributing to a deeper understanding of such development in relation to careers in nursing leadership.

## 1 Introduction

Successful nursing leadership requires competent communication. According to recent research, nurse leaders' competent communication is related to nurses' performance (Ibrahim and Ahamat 2019), job satisfaction (Fowler et al. 2021) and career length (Nurmeksela et al. 2023). Relatedly, competent communication also plays a critical role in preventing burnout and promoting nurse retention (Jankelová and Joniaková 2021; Stillman et al. 2024; Tang and Hudson 2019). However, despite its recognised importance, comparatively little is known about how nurse leaders develop communication competence over time and within complex organisational contexts. Therefore, this study describes nurse leaders' perceptions of the development of their own interpersonal communication competence and examines factors that hinder or promote it.

## 2 Background

Defining communication competence is the essential first step in understanding its development. At the interpersonal level, communication competence can be examined from two perspectives: interpersonal and relational communication competence (Hannawa and Spitzberg 2015). Interpersonal communication competence focuses on an individual's capacity to achieve personal goals in interpersonal communication settings (Spitzberg 2013), while relational communication competence emphasises building and sustaining meaningful relationships (Hannawa and Spitzberg 2015).

Following the communication literature, this study has focused on interpersonal communication competence, which is commonly defined by the effectiveness and appropriateness of behaviour within a given context. Relational competence is considered here as a component of interpersonal communication competence, thus highlighting its relational dimension. (Hannawa and Spitzberg 2015) Competent communication is typically evaluated based on two criteria: its effectiveness, how well communication achieves its intended goals, and its appropriateness, how well it aligns with social norms and expectations (Hargie 2019; Horila 2019). Although interpersonal communication skills and competence are frequently used interchangeably, competence is usually regarded as a broader concept that includes knowledge, skills and motivation (Hargie 2019).

Previous studies have demonstrated that nurse leaders' interpersonal communication competence consists of ethical principles, message competence, relational competence and task competence (Kämäräinen et al. 2024, 2025). According to a conceptual framework introduced in recent research, nurse leaders' message competence encompasses the basic communication skills that are critical to nursing leadership, such as active listening and creating messages. Relational competence, meanwhile, refers to the competence needed in building, nurturing and maintaining good professional relationships, while task competence concerns the interpersonal communication competence that enables the achievement of tasks and goals related to nursing leadership (Kämäräinen et al. 2024). Lastly, ethical principles entail the fundamental ethical principles of communication, such as truthfulness and equality, that should be aspired to in all communication (Kämäräinen et al. 2025).

From the perspective of development, interpersonal communication competence has been conceived as a combination of inherent and learned abilities. Despite the significant influence of genetic inheritance, studies have found that the link between competence and evolutionary and biological factors is indirect at best. (Hannawa and Spitzberg 2015) Instead, the ability to learn and develop has been seen as an essential aspect of interpersonal communication competence. In other words, such competence has been seen as socially defined and constructed; although people may be innately capable of communicating and developing language, the use of skills in ways that are considered skilful depends on social perceptions, including perceptions of the appropriateness and effectiveness of communication (Hannawa and Spitzberg 2015; Hargie 2019).

In a lifespan perspective, interpersonal communication competence is seen as a lifelong, multifaceted and multidirectional process in which skills can both improve and diminish (Fisher and Roccotagliata 2017). Development occurs within individuals, between individuals and through interaction with one's environment (Fisher and Roccotagliata 2017), aligning with social cognitive theory (Ahsan 2024). According to the theory, learning occurs through the dynamic and complex interplay of cognitive, behavioural and environmental factors. This viewpoint posits that individuals can influence their thinking processes, motivations, and behaviours and thus have the power to influence the changes that occur in themselves. By clarifying the underlying processes of individual learning, it emphasises the significant impact of social influences on behaviour and underscores the importance of self‐efficacy in enhancing learning and performance (Ahsan 2024).

A previous systematic literature review examined nurse leaders' interpersonal communication competence at different skill levels. The study identified novice, competent and expert levels (Kämäräinen et al. 2024), echoing Benner's (2001) stage model of competence in nursing practice, which emphasises the importance of experiential learning and the practical application of theoretical knowledge for developing expertise. The available evidence partly supported the view that job role and work experience may impact the development of nurse leaders' interpersonal communication competence and emphasised the importance of lifelong learning and communication training. However, the description focused primarily on novice‐level skills and failed to describe the interrelationships between competencies or the complexity of competence development (Kämäräinen et al. 2024).

The recent review on nursing leadership competencies underscores the need for highly developed communication skills among nurse leaders. Overall, exploring competencies demonstrated a great need for relational competence, including the ability to build and maintain good relationships and manage conflicts (Rojko et al. 2025). Researchers have also examined the development of discrete interpersonal communication skills, such as nurse leaders' conflict resolution skills and interviewing skills (Seabold et al. 2020) and collaboration skills (Servick et al. 2023). Extant studies have focused largely on intervention designs, which have limited our understanding of how nurse leaders' interpersonal communication competence develops. Therefore, this study addresses this gap by exploring the development of interpersonal communication competence among nurse leaders from their own perspective, with particular attention. By pursuing this avenue of inquiry, this study sought to increase understanding of the factors that inhibit and contribute to the development.

## 3 The Study

The aim of the study was to describe nurse leaders' perceptions of factors related to the development of their own interpersonal communication competence.

The two main research questions were as follows:

1. How do nurse leaders perceive the development of their own interpersonal communication competence?
2. What factors are related to the development of nurse leaders' interpersonal communication competence?

## 4 Methods

### 4.1 Design

A descriptive qualitative design was chosen to capture nurse leaders' subjective perspectives on interpersonal communication competence within their professional context, aligning with the principles of naturalistic inquiry. This approach allows for a nuanced data‐driven interpretation of participants' experiences, aiming to produce a thorough description of the meanings these experiences hold for those involved (Villamin et al. 2025). Reporting follows the standards for reporting qualitative research (SRQR) checklist (O'Brien et al. 2014).

### 4.2 Study Setting and Recruitment

The data for this study was originally collected as part of a larger interview study of nurse leaders' interpersonal communication competence. While the previous sub‐study described the structure and content of nurse leaders' interpersonal communication competence (Kämäräinen et al. 2025), this study addressed the research question on the development of nurse leaders' interpersonal communication competence.

The data was collected from nurse leaders in three wellbeing service counties in Finland using purposive sampling. In Finland, 21 wellbeing service counties are regional public entities established in 2023 as part of a structural health and social services reform. Under this reform, the organisation and governance of public healthcare, social welfare and rescue services were integrated under a unified administrative structure and budget. The counties are governed by elected councils and funded by the central government. The wellbeing service counties selected for this study represented various geographical areas and organisational sizes, each with a distinct nursing leadership structure. Nurse leaders in the selected counties manage nursing practices and lead nursing staff in both the health system and social welfare services.

The inclusion criteria included nurse directors, nurse managers and nurse leaders with equivalent titles in healthcare and social welfare services who were acknowledged as competent communicators within their organisations. To ensure diversity in leadership perspectives, participants were recruited from various levels of nursing management (unit‐level, middle management and top‐level management) and from a range of organisational contexts, including specialised medical care, primary health care and social care services (home care). The main inclusion criterion was holding a nursing leadership position; consequently, participants were not required to have a formal educational background in nursing. As communication literature describes interpersonal communication competence as a relational and contextual phenomenon, the inclusion criterion for identifying competent communicators was based on the chief nursing officer's subjective assessment of each employee's interpersonal communication competence. Before sending out the invitation letter, the chief executive nursing officers of the wellbeing services counties were informed about the aim of the study, the study group and the subjectivity of competence assessment. They then forwarded the invitation to the nurse leaders they identified as potential participants in the study.

### 4.3 Data Collection

Twenty‐one nurse leaders (*n* = 21) consented to participate in the study. Individual semi‐structured interviews were conducted between February and April 2024. Interviews were carried out face‐to‐face and remotely by the first author, according to the preference of the nurse leader. Interviews were recorded remotely via video or in person via audio. As the analysis concentrated on the spoken content and its underlying meanings, audio and video conferencing were considered suitable methods of data collection that preserved the quality of the data. The interview guide is presented in File S1.

Data saturation was reached after the 19th interview, at which point no new information emerged from the data. Based on the interviewer's observations, the final two interviews confirmed and supplemented the previously collected data. The first author transcribed the recordings into written form using the Microsoft transcription tool in the Teams and Word software (Microsoft 2024). The total number of transcription pages was 103 (Times New Roman, 12‐point font, single‐spaced). All transcriptions were confirmed by the first author and corrected to be consistent with the original recordings. Accordingly, personal details were deleted from the transcriptions to ensure anonymity.

### 4.4 Data Analysis

The data were analysed using an inductive content analysis. This data‐driven process involved identifying answers to the research questions within the dataset, which were then organised into categories at varying levels of abstraction and interpretation. (Graneheim et al. 2017; Vears and Gillam 2022) The analysis started with a thorough examination of the interview data to create an accurate overview. Coding began by identifying and marking meaning units emerging from the data. The unit of analysis consisted of phrases, sentences, or multi‐sentence ideas that reflected important themes and phenomena. All meaning units were collected into a file and anonymised.

**FIGURE 1:**
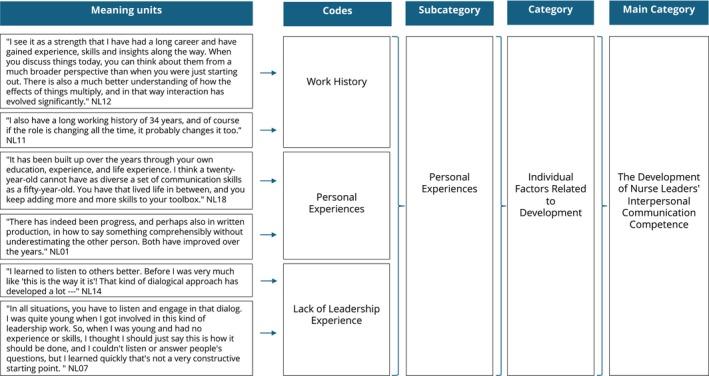
Example of analysis process.

In the following phase, each meaning unit was coded and descriptively named. After all units were coded, they were organized into appropriate subcategories by combining similar codes that represented the same category. Then the subcategories were combined into higher categories representing categories and the main category. The inductive analysis concluded when all codes were classified and the structure of the analysis was clear. An example of the analysis process is illustrated in Figure 1.

The initial coding and categorisation were conducted by the first author using ATLAS.ti software. During the collaborative analysis process, the categorisations and naming of categories generated by the analysis were discussed regularly within the research team. As the interviews were carried out in Finnish, the analysis was initially carried out in Finnish and then translated into English. To ensure that all meanings were preserved during the translation process, the accuracy of the translation was verified against the originals using a back translation into Finnish. The data analysis resulted in 989 open codes, which were classified into two categories and eight subcategories.

### 4.5 Ethical Considerations

Permission to conduct the study was given by all participating wellbeing service counties. The study adhered to the ethical principles outlined by the Finnish National Board on Research Integrity TENK (Finnish National Board on Research Integrity 2019) and complied with the EU General Data Protection Regulation (Regulation (EU) 2016/679; European Commission 2018). According to Finnish legislation and TENK guidelines, ethical approval was not required from the research organisation. The research topic did not involve sensitive issues. The participants were experts in the field, and the interviewer possessed expertise in the research area. Potential risks relating to confidentiality and emotional discomfort were considered and mitigated through careful study design and informed consent procedures.

The invitation letter covered the study's purpose, data management procedures, confidentiality and de‐identification and participants' right to retract their consent at any time. After reviewing the letter, participants gave their verbal consent to participate directly to the first author. This verbal consent was audio‐recorded at the beginning of each interview session. All direct personal data have been removed from the transcripts, and the nurse leaders are identified by numbers 1–21. The recordings containing consent statements and the code key file have been stored securely. Participant details are reported in total, and their respective fields or units have been omitted to ensure confidentiality.

### 4.6 Rigour and Reflexivity

Reflexivity (Korstjens and Moser 2018), confirmability, credibility, authenticity, transferability and dependability (Connelly 2016) were used as the criteria to assess the trustworthiness of the study. Considering reflexivity, the research team consisted of two members with PhDs in nursing science, one with a PhD in communication science, and a doctoral researcher. Combining diverse backgrounds in communication and nursing science enriched the team's capacity to critically evaluate and interpret the phenomena (Korstjens and Moser 2018). The first author, the doctoral researcher, conducted all the interviews. Throughout the research process, the research team regarded participants as important sources of information about a topic (Korstjens and Moser 2018). The first author conducted the analysis in close collaboration with the research team, which strengthened the confirmability of the study. The multidisciplinary expertise of the team further enabled a deep understanding of the research topic, which also contributed to the credibility and confirmability of the study (Connelly 2016).

To enhance credibility and authenticity, the research process has been systematically and accurately reported (Kyngäs et al. 2020). Furthermore, original expressions were chosen to describe the data in the most illustrative way to increase the reporting's authenticity and confirmability (Connelly 2016).

## 5 Findings

### 5.1 Participant Characteristics

A total of 21 nurse leaders participated in the study. Most of the participants were female (*n* = 17) and worked in mid‐level or top‐level nursing leadership positions (*n* = 12), with nine working as nurse managers. Most of them had an educational background as registered nurses (*n* = 17). The remaining four had social and healthcare education from the university or the University of Applied Sciences, and their qualifications enabled them to work in nursing leadership positions within the Finnish system. The average age of the participants was 52 years, with a range of 38 to 62 years. Their average experience in nursing leadership was 15 years, with a range of five to 24 years. Two participants reported having previously received communication training.

### 5.2 Nurse Leaders' Perceptions of the Development of Interpersonal Communication Competence

Nurse leaders perceived the development of interpersonal communication competence as a lifelong and continuous process related to two categories: individual factors and interpersonal factors. Eight subcategories were identified in the descriptions of nurse leaders related to the development of interpersonal communication competence. An overview of the results is presented in Table 1.

**TABLE 1:** Overview of the results of nurse leaders' interpersonal communication competence development and factors promoting (+) and hindering (−) development.

Main category	Category	Subcategory	Factor related to development (+/−)
The development of nurse leaders' interpersonal communication competence	Individual factors related to development	Inherent qualities	+ Strengthening inherent qualities – Inhibitory inherent traits
		Personal experiences	+ Work history + Personal experiences – Lack of leadership experience
		Reflexivity	+ Self‐reflection + Self‐evaluation
		Motivation	+ Motivation to communicate + Motivation to self‐development + Feedback‐orientation – Low motivation to self‐development
		Communication training	+ Sufficient communication training in the background education + Continuing education + Self‐study – Lack of training
	Interpersonal factors related to development	Situational contexts	+ Diverse encounters + Situation‐based examples
		Feedback	+ Sufficient feedback – Lack of feedback
		Social support	+ Counselling + Co‐reflection

All participants described how their interpersonal communication competence had improved throughout their careers. The nurse leaders' perceptions regarding their own competence development are presented below. The perceptions are organised by the main categories and subcategories related to competence development, beginning with individual factors.

### 5.3 Individual Factors Related to Development

Individual factors refer to factors in the nurse leaders themselves that either promoted or hindered the development of their interpersonal communication competence.

#### 5.3.1 Inherent Qualities

Inherent qualities, such as personality, were seen as having a direct impact on interpersonal communication competence. Some nurse leaders viewed interpersonal communication competence as partly inherent, “given” qualities, while others believed certain inherent traits could even hinder the development of competence. Overall, nurse leaders considered some aspects of interpersonal communication competence to be inherent and thus somewhat immutable. However, for the most part, competence was seen as a learned and evolving phenomenon, as described by the following nurse leader: “You can't change your personality completely, but you can change certain behaviors in yourself.” NL07.

#### 5.3.2 Personal Experiences

Personal experiences, particularly successes and failures, greatly contributed to the development of nurse leaders' interpersonal communication competence. According to the nurse leaders, interpersonal communication competence is developed largely through “daily practice” and “experience.” Furthermore, participants described how the required interpersonal communication competence varied by job position and work environment and how individual work history has thus greatly influenced its development.

Conversely, nurse leaders recognised that a lack of personal experience hindered the development of interpersonal communication competence. This observation was reflected in participants' descriptions of their early career failures in communication, particularly when they compared their current interpersonal communication competence to when they started working as a nurse leader.

The successful implementation of tasks and changes in nursing leadership had particularly developed the influencing skills of the nurse leaders, and participants described numerous valuable lessons learned from these experiences. One nurse leader described how vision and goals had become more prominent in their communication. On the other hand, career advancement had opened new opportunities that further developed their influencing skills and helped them achieve their goals. Participants also noted the importance of challenging communication situations, where the likelihood of success increased with experience. The role of personal experiences in the development of communication competence was illustrated as follows:

When different situations arise and you handle them, you see how it should have been done … of course, that supports you. Then you also gain confidence that this is the way I should do it to succeed. It develops through experience, and you gain confidence, and you develop your own style. NL04

In contrast, situations that occurred only once or twice in a career were seen as problematic for development:

Those rare challenging situations are the ones where you don't really know how to act as a leader. You may only have one in your entire leadership career, so you don't get a chance to develop your skills. You handle it based on how well you were prepared. NL10

#### 5.3.3 Reflexivity

Nearly all participants emphasised the importance of self‐reflection in promoting the development of interpersonal communication competence. Nurse leaders described critically examining their own thought processes and communication behaviours, as well as the communication situations themselves and the emotions they aroused:

I am also ready to examine it [own communication], and I can critically see and consider new perspectives, and change a certain aspect. NL05

In addition, the nurse leaders noted that they continuously evaluate their own interpersonal communication competence, described as “critical examination,” “standing in front of a mirror,” and “self‐evaluation.” This process has required a clear understanding of oneself as a communicator, or a “self‐concept,” which means an understanding of one's own communication style, strengths, and areas for improvement. According to the nurse leaders, this self‐concept also developed over time, leading to a more extensive communicator image.

#### 5.3.4 Motivation

The pivotal role of motivation for communication and self‐development was underlined in developing interpersonal communication competence. As their interpersonal communication skills improved, the nurse leaders became more motivated to communicate, especially to use communication as a leadership tool. Participants explained how they had learned not to take feedback personally and how their feedback orientation, or their “openness to feedback,” had also improved over the years; they began to see feedback as essential to the development of interpersonal communication competence:

I have had to metaphorically stand in front of a mirror and face it and think, ‘Hey, did I really act like this? My goodness, I really need to improve my behavior.’ I am grateful for the feedback I have received; it has taught me a lot. NL09

However, some nurse leaders described that development had been partially halted over their long careers. These participants referred to themselves as “fixed in their habits” and were no longer able to motivate themselves to actively develop their skills. Notably, nurse leaders also acknowledged that skills and motivation were not only developed but also lost over the years of their careers. For example, one nurse leader described how their enthusiasm for communication had diminished over a long career: “Maybe when I was younger, I was more enthusiastic about presenting things because I didn't know that I had to be a little more careful. In some ways, maybe that kind of enthusiasm has diminished a little bit.” NL02.

#### 5.3.5 Communication Training

The nurse leaders noted that receiving sufficient communication training, whether as part of their background education or through continuing education, has promoted the development of their interpersonal communication competence. According to the participants, communication training and theoretical knowledge were vital facets of successfully reflecting on their own communication. They supplemented such formal education and training with self‐study on specific aspects of communication. In addition, skills were trained, and new ways of communicating and interacting were experimented with. The importance of receiving feedback when practising new skills was also emphasised: “Good communication requires practice and receiving feedback.” NL20.

In general, however, the amount of communication training was considered insufficient, and participants expressed a need for more training.

There is still no training available at the organizational level for this matter, but then you have to learn on your own. NL17

### 5.4 Interpersonal Factors Related to Development

Interpersonal factors refer to relational factors within the nurse leaders' professional environment that either promote or hinder the development of interpersonal communication competence.

#### 5.4.1 Situational Contexts

The nurse leaders described a variety of situational contexts that facilitated the development of their interpersonal communication competence. These contexts encompassed specific encounters in various work situations, organisational communication settings and personal contexts. One of the most frequently cited was organisational communication environments in which development took place through various meeting situations and face‐to‐face interaction. Furthermore, such development was facilitated by the use of various communication channels, which required written communication skills and the adoption of new communication methods.

In general, nurse leaders perceived their own and their colleagues' interpersonal communication competence as good, attributing its development largely to the high level of competence required in the nursing leadership profession. They stressed that a notable driver of their development was the evolving situational contexts in which they worked, particularly changes in work and communication environments. These changes included, for example, changes in physical work environments, organisational structures, and leadership styles over time. Regarding changes in the organisational communication environments, particular attention was drawn to the explosion in the use of remote communication technologies during the COVID‐19 pandemic, which required nurse leaders to upgrade their skills and “technical know‐how”:

The first thing that comes to mind is all these technical skills for Teams and email communication. If I think about the early stages of my leadership career, those methods were hardly used for communication. In hospitals, we used a lot of paper weekly newsletters to communicate. It sounds funny now, but it wasn't that long ago that we still used them. NL16

Furthermore, all participants viewed learning from situational contexts in their professional environments as important. Both positive and negative examples of communication were influential learning experiences in these contexts. Specifically, good role models enhanced nurse leaders' skills in areas such as creating messages, regulating their own communication and supporting workplace communication. Conversely, some nurse leaders felt that individuals who previously held their positions had served as poor role models, and evaluating these poor examples strengthened their role‐acting competence and self‐conception as communicators. Other negative examples included failed communication situations or situations where communication was perceived as inappropriate or counter to their values. As the following nurse leader described, situational learning experiences most often involved peers, senior leaders and direct managers, but others also played a role: “I always say that my role models in leadership are my father, who was a business executive and raised me to be such a skilled communicator, doing a pretty good job at it, and my former manager.” NL21.

Alongside work and communication environments, the development of interpersonal communication competence was supported by personal contexts, including close relationships and social activities.

#### 5.4.2 Feedback

Participants considered receiving feedback as critical to developing interpersonal communication competence. Positive feedback “felt good,” and it encouraged and reinforced specific communication behaviours. Constructive feedback, which participants deemed “good feedback,” was also perceived as necessary for development. Despite the importance of receiving feedback, nurse leaders reported receiving it infrequently. In addition to the feedback given, they also described how they observed feedback, such as audience's nonverbal responses, in communication situations, to evaluate the success of their communication. The partly unconscious developing process of developing skills through receiving feedback was described as follows:

Throughout my career, I have received feedback that I should give more feedback. I started thinking that I didn't know how to give feedback, but somewhere along the way, I began receiving feedback that I actually did know how to give it. NL01

#### 5.4.3 Social Support

Lastly, the nurse leaders relayed the meaningful role of counselling and co‐reflection in the development of their interpersonal communication competence. Sharing experiences along with receiving advice and guidance from others, such as more experienced leaders or colleagues, was seen as crucial to the development of communication competence: “The personnel director said during tough discussions, which I found comforting, that keep that—humor is allowed, as it can lighten things up in certain situations. However, as I mentioned earlier, it's a skill.” NL11.

Participants viewed co‐reflection as a collaborative process in which they discussed communication situations with others and reflected on their practices and experiences. As the following quotation illustrates, this process also could involve mutual support and shared insights to enhance personal development: “It is good that we have a common workspace where we head nurses work so we can discuss these issues. Especially when you are new to this job, you get concrete support and advice from there.” NL16.

## 6 Discussion

This study has outlined nurse leaders' perceptions of the development of their own interpersonal communication competence and the factors they considered important in this development. According to their responses, the development of nurse leaders' interpersonal communication competence is a lifelong and dynamic process comprising both individual and interpersonal factors, a finding that is consistent with the previous literature (Fisher and Roccotagliata 2017; Hannawa and Spitzberg 2015; Hargie 2019). The study has also provided novel insight into how nurse leaders perceived the development of their own interpersonal communication competence throughout their careers.

By identifying factors related to the development of nurse leaders' interpersonal communication competence, this study has demonstrated the importance of informal learning in workplaces. At the same time, nurse leaders expressed a great need for formal education. According to the participants, they had received relatively little communication training, which may have contributed to their emphasis on its importance. Relatedly, evidence from other studies has revealed the positive impact of continuing education on nurse leaders' communication (Kuraoka 2018; Seabold et al. 2020; Servick et al. 2023). For example, Kuraoka (2018) reported self‐assessed improvement in nurse leaders' communication following leadership training, while Servick et al. (2023) found a statistically significant positive effect on nurse leaders' communication skills at a one‐year follow‐up. Similarly, Seabold et al. (2020) found an increased recognition of the importance of communication‐related competence knowledge at a one‐year follow‐up. Taken together, the findings from this and prior studies suggest that investing in communication training may be a relevant strategy to enhance nursing leadership outcomes.

Participants also highlighted the importance of social support and feedback in developing their interpersonal communication competence. This finding is consistent with the social cognitive learning theory, which asserts that a positive environment and support from a reliable source are two of the main sources of self‐efficacy (Huang et al. 2025; Yu and Zhou 2025). In an experiential intervention study of young women's leadership by Laritza et al. (2025), participants saw mentors as important to their professional development by inviting them to examine themselves and consider their ideas and beliefs. Other studies have found that feedback helps leaders understand their current competencies and whether their communication is achieving its goals (Emam et al. 2024; Lacerenza et al. 2017). In their study, Emam et al. (2024) found that collecting a wide range of feedback from staff, peers and leaders provided a comprehensive view of nurse leaders' strengths and areas for development, enhanced their self‐conception and improved their leadership performance. Regarding the importance of feedback for development, participants in this study considered the amount of feedback they had received to be relatively low. Combined with the reported lack of training, this may suggest that the development of leaders' communication competence is not sufficiently prioritized in contemporary healthcare organisations. This interpretation is supported by studies reporting a lack of leadership training in many nursing leadership roles (Emam et al. 2024). Furthermore, the discrepancy between research evidence emphasizing the importance of these methods for professional development and current practices may indicate systemic challenges in translating evidence into leadership practice. This highlights the need for strategies to integrate evidence‐based communication competence development into leadership education and organisational policy frameworks. As relational leadership approaches become more prominent, the development of nurse leaders' interpersonal communication competence is no longer merely important but has emerged as a critical priority for educational and healthcare institutions.

Overall, this study highlighted the need for various practices to promote the development of nurse leaders' interpersonal communication competence. The need for diverse practices is demonstrated, for example, by the nurse leaders' perception of the need for education and knowledge to reflect their own communication. The need for active reflection in learning is also emphasised in constructivist learning theory, where development has been seen as occurring through training different leadership competencies and reflecting on these experiences, as well as confronting and solving problems in practice environments (Lacerenza et al. 2017). According to the literature review, reflective learning is common in nursing leadership development programs, alongside didactic, interactive and experiential learning (Ullrich et al. 2021). However, this study does not describe which practices were found to be most relevant to competence development, and therefore future research should also explore the importance of different methods for the development of interpersonal communication competence.

The findings indicated that all dimensions of interpersonal communication competence theory (Hannawa and Spitzberg 2015; Spitzberg 2013) were reflected in the participants' descriptions. For example, nurse leaders in this study described the need for knowledge and understanding (cognitive), as well as the development of the skills through various ways (behavioural) and the essential motivation for promoting development (affective). Moreover, the categories of nurse leaders' interpersonal communication competence identified in Kämäräinen et al. (2024) can be seen in this study, highlighting that development occurs widely in all competence areas. In addition, the study can be seen as encompassing interpersonal communication competence as both a relational and situational phenomenon. These findings may support the view that communication skills training should not focus on teaching micro‐level skills but should be developed in a broader context. Previously, training focused on specific skills has been criticised for making it difficult to learn a single component without understanding the meaningful entities. According to this approach, communication skills are not used in complete isolation from other skills (Hargie 2019). It was demonstrated in this study, for example, by nurse leaders' perceptions that development occurred broadly across various areas simultaneously, partly as an interplay with other skills. The importance of the interrelationships between skills in interpersonal communication training has also been highlighted in previous literature (Hargie 2019).

In summary, nurse leaders perceived the development of their interpersonal communication competence as a continuous and lifelong process. An examination of promoting and hindering factors revealed that the development of nurse leaders' interpersonal communication competence was facilitated by the interplay of individual and interpersonal factors, encompassing various competence categories and dimensions. In addition, the study demonstrated that interpersonal communication competence is a relational and situational phenomenon. Therefore, there is a need to develop holistic communication interventions for nurse leaders that account for the interrelationships between competencies, e.g., relational competence and task competence. Furthermore, such interventions should combine diverse practices of developing interpersonal communication competence rather than focusing on a single method. Alongside these efforts, it is also important to establish different opportunities for nurse leaders to develop their interpersonal communication competencies in their work.

### 6.1 Strengths and Limitations

The study has various strengths and limitations. The qualitative research design provided valuable insights into the development of nurse leaders' interpersonal communication competence in a real‐world setting. The participants' descriptions of the development of their interpersonal communication competence were rich, and the study reached the saturation point of data. However, participants were not asked for feedback on the findings, which may have influenced the validity of the researchers' inferences from the data. Additionally, the study did not report detailed background information on the participants, which may undermine the transferability of the results. Lastly, the research was conducted in three Finnish public social and healthcare organisations, and the results may not be transferable to healthcare systems in other countries.

### 6.2 Recommendations for Further Research

Overall, the findings underline several avenues for future inquiries. First, they indicate the necessity of conducting further research focused on promoting nurse leaders' interpersonal communication competence. They also emphasise the need to explore different methods in promoting interpersonal communication competence. In this context, conducting intervention development studies involving nurse leaders and other relevant stakeholders in a co‐design intervention could be useful. Lastly, an exploration of the differences in the interpersonal communication competence required for various positions and areas in nursing leadership would help deepen our understanding of such competence and its various applications and dimensions.

### 6.3 Implications for Policy and Practice

This study's findings demonstrate the importance of diverse competence development practices in today's workplaces. More specifically, they reveal how crucial it is for healthcare organisations to provide nurse leaders with more opportunities to develop their interpersonal communication competence in their work, both through formal communication training and their daily work activities. In addition, to reinforce these efforts, educational institutions should prioritise and increase the amount of interpersonal communication training in nursing leadership programs.

## 7 Conclusions

This study has provided valuable insights into the development of nurse leaders' interpersonal communication competence, contributing to a better understanding of its development throughout these individuals' careers. The findings show that nurse leaders' interpersonal communication competence develops as a result of an effective interplay of various individual and interpersonal factors. Overall, the study has highlighted the value of various practices in developing interpersonal communication competence in workplaces. Concurrently, the findings also emphasised the importance of communication training in nursing leadership education and continuing education. The findings also suggest that nurse leaders' interpersonal communication competence evolves throughout their careers, and there are interrelationships between the development of interpersonal communication competencies. Therefore, holistic interventions to cultivate interpersonal communication competence, in addition to micro‐skills development programs, are needed in the future. Alongside this, it is also important to implement different opportunities for nurse leaders to develop their skills while in their roles.

## Author Contributions

P.K., L.M., A.N., and T.K. made substantial contributions to conception and design, or acquisition of data, or analysis and interpretation of data. P.K., L.M., A.N., and T.K. involved in drafting the manuscript or revising it critically for important intellectual content. P.K., L.M., A.N., and T.K. given final approval of the version to be published. Each author should have participated sufficiently in the work to take public responsibility for appropriate portions of the content. P.K., L.M., A.N., and T.K. agreed to be accountable for all aspects of the work in ensuring that questions related to the accuracy or integrity of any part of the work are appropriately investigated and resolved.

## Ethics Statement

According to the ethical regulations governing Finnish research, ethical review was not required (University of Eastern Finland (UEF) Research Ethics Committee/Description No 33/2024).

## Consent

The authors have nothing to report.

## Conflicts of Interest

The authors declare no conflicts of interest.

## Supporting information


**Data S1:** Standards for Reporting Qualitative Research (SRQR)*.

**File S1:** Semi‐structured interview guide.

## Data Availability

The data that support the findings of this study are available on request from the corresponding author. The data are not publicly available due to privacy or ethical restrictions.
