# Supplementary material for: Nurse Leaders' Perceptions of Development of Their Own Interpersonal Communication Competence: A Qualitative Descriptive Study in Social and Healthcare Organisations
Source: J Adv Nurs. 2025 Oct 15;82(6):6504–14. doi: 10.1111/jan.70281 (PMC13176672; doi:10.1111/jan.70281)
Supplement: Supplementary file 2 — File S1: Semi‐structured interview guide. [file JAN-82-6504-s002.pdf]

Nurse Leaders' Perceptions of Development of Their Own Interpersonal Communication Competence: A Qualitative Descriptive Study in Social and Healthcare Organizations

**Supplementary Material 1.** Semi-structured interview guide.

**Background information**

- Age, gender, education, organization, job title, work experience, communication training background, the assessment of the current stage of their own interpersonal communication competence

**Theme 1:** The development of interpersonal communication competence

- Perceptions and experiences related to:
  - the development of their own interpersonal communication competence during their career
  - the role of inherent factors on interpersonal communication competence
  - the interpersonal communication competence as an evolving phenomenon
  - developmental areas of interpersonal communication competence.

*Examples of Questions*

- "How do you think your interpersonal communication competence has developed during your career?
- "To what extent do you think your personality is reflected in your interpersonal communication competence?"
- "In which area of your interpersonal communication competence do you feel you have improved the most?"
- "How has your perception of yourself as a communicator developed during your career?"

**Theme 2:** The factors related to the development of interpersonal communication competence

- Perceptions and experiences related to different methods to develop interpersonal communication

*Examples of Questions*

- "How have career development and different roles changed or developed your interpersonal communication competence?"
- "How have your previous experiences influenced the development of your interpersonal communication competence?"
- "How have changes in the work environment developed the way you interact with others?"
- "How do you think the feedback you have had has contributed to your development?"

**Final question**

- Free thoughts and additions on the themes

*Examples of Questions*

- "Do you have anything to add to this theme?"
